# Differential transcriptome analysis supports *Rhodnius montenegrensis* and *Rhodnius robustus* (Hemiptera, Reduviidae, Triatominae) as distinct species

**DOI:** 10.1371/journal.pone.0174997

**Published:** 2017-04-13

**Authors:** Danila Blanco de Carvalho, Carlos Congrains, Samira Chahad-Ehlers, Heloisa Pinotti, Reinaldo Alves de Brito, João Aristeu da Rosa

**Affiliations:** 1 Department of Parasitology, São Paulo State University (UNESP), School of Pharmaceutical Sciences, Araraquara, São Paulo, Brazil; 2 Department of Genetics and Evolution, Federal University of São Carlos (UFSCar), São Paulo, Brazil; Onderstepoort Veterinary Institute, SOUTH AFRICA

## Abstract

Chagas disease is one of the main parasitic diseases found in Latin America and it is estimated that between six and seven million people are infected worldwide. Its etiologic agent, the protozoan *Trypanosoma cruzi*, is transmitted by triatomines, some of which from the genus *Rhodnius*. Twenty species are currently recognized in this genus, including some closely related species with low levels of morphological differentiation, such as *Rhodnius montenegrensis* and *Rhodnius robustus*. In order to investigate genetic differences between these two species, we generated large-scale RNA-sequencing data (consisting of four RNA-seq libraries) from the heads and salivary glands of males of *R*. *montenegrensis* and *R*. *robustus*. Transcriptome assemblies produced for each species resulted in 64,952 contigs for *R*. *montenegrensis* and 70,894 contigs for *R*. *robustus*, with N50 of approximately 2,100 for both species. SNP calling based on the more complete *R*. *robustus* assembly revealed 3,055 fixed interspecific differences and 216 transcripts with high levels of divergence which contained only fixed differences between the two species. A gene ontology enrichment analysis revealed that these highly differentiated transcripts were enriched for eight GO terms related to AP-2 adaptor complex, as well as other interesting genes that could be involved in their differentiation. The results show that *R*. *montenegrensis* and *R*. *robustus* have a substantial quantity of fixed interspecific polymorphisms, which suggests a high degree of genetic divergence between the two species and likely corroborates the species status of *R*. *montenegrensis*.

## Introduction

Chagas disease is a parasitic infection distributed through the Neotropical region. Its etiologic agent is the protozoan *Trypanosoma cruzi*. The main mode of transmission to humans is vectorial, and it is estimated that between six and seven million people are infected worldwide [1]. Triatomines, the vectors of Chagas disease, are exclusively haematophagous during their five nymphal instars and as adult males and females [2]. These vectors are part of the subfamily Triatominae (Hemiptera:Reduviidae), which comprises of five tribes [2, 3]. The tribe *Rhodniini* consists of two genera: *Psammolestes*, which includes three species, and *Rhodnius* containing 20 species, which are found in the Americas [4–6]. The genus *Rhodnius* has a wide distribution in most of Latin America, with the exception of Argentina, Chile, Uruguay, and Paraguay [2, 4, 7], consisting of tree-dwelling species that use as their main habitat palm tree canopies that shelter mammals. The genus is therefore considered sylvatic [2, 4, 7], but many *Rhodnius* species are adapting to peridomiciliary and even intradomiciliary environments as a result of the changes to their natural habitats that have been caused by urbanization [8, 9]. For example, whereas *Rhodnius prolixus* has been found in rural housing all over Latin America [10] *Rhodnius stali* has been reported to colonize housing in indigenous communities [11, 12]. *Rhodnius neglectus* has also been found in urban centers far from endemic areas [13, 14]. Therefore, those reports suggest the possibility of Chagas disease transmission by *Rhodnius* species in domestic environments, whether directly by the vector or indirectly through food contaminated by infected triatomine feces [15].

The identification of species of the genus *Rhodnius* has been largely based on the observation of at least 19 morphological traits [2, 5]. Even when these features are used, the morphological distinction between *Rhodnius* species still presents difficulties [16–18]. Examples of specific similarities in the genus *Rhodnius* are reflected in the description of *R*. *stali* [19], which has been grouped with *R*. *pictipes*, as well as the description of *R*. *montenegrensis* [17], which was incorrectly identified as *R*. *robustus*. For the description of *R*. *montenegrensis* [17], the authors evaluated previously used morphological traits, classic and geometric morphometry, and a phylogenetic analysis of a portion of the mitochondrial cytochrome b gene [16].

In the search for a way to identify closely related species, many authors have resorted to molecular data such as the use of mitochondrial and nuclear DNA marker analyses [20]. However, new methodologies have provided a wider range of possibilities to address this issue [21, 22]. RNA-seq analysis, for example, allows for the identification of polymorphisms in thousands of different transcripts in a faster and much more cost effective way relative to individual gene analysis or genome studies in non-model organisms [23]. Transcriptomes comprise all transcripts that are expressed in a specific tissue and developmental phase, several of which that may play a functional role in the organism; therefore, they may reflect in part phenotypic variation and population differentiation. One interesting aspect of the analysis of transcriptomes among closely related species is the possibility of identification of expressed genes that may have evolved under positive selection, which could be more differentiated, and even involved in the species divergence [24].

The main goal of this study was to search for fixed differences between *R*. *montenegrensis* and *R*. *robustus* in thousands of transcripts expressed in the cephalic transcriptome of males of both species. The findings showed a substantial number of fixed differences that support the hypothesis that these are genetically distinct species. Hence, the genetic differentiation found in this study confirms the specific status of *R*. *montenegrensis* previously reported by Rosa *et al*. [16].

## Methodology

### Sampling, dissection, and RNA extraction

The individuals of the species used in this study have been maintained at the Triatominae Insectarium of the Department of Biological Sciences of the Faculty of Pharmaceutical Sciences, Unesp, Araraquara, São Paulo, Brazil in individually identified glass crystallizers with internal divisions. The specimens are fed on ducks on a monthly basis. Male specimens were separated as fifth instar nymphs. Once they reached the adult phase, they were blood-fed on ducks twice in the first week and not fed seven days before the dissection. Male heads were carefully removed via cervical detachment in order to keep the salivary glands intact. It is important to note that compound eyes were removed from the specimens prior to RNA extraction due to the large quantity of pigments that the reticular cells of these insects exhibit.

Total RNA was extracted using three sets of heads and their respective salivary gland pairs based on a modified version of the TRIzol/chloroform protocol [25], followed by differential precipitation with lithium chloride [26]. Finally, RNA samples were quantified in Qubit and their purity was measured using the standard ratios 260/230 and 260/280 in NanoDrop and visual inspection of RNA bands after agarose gel electrophoresis.

### RNA sample preparation and sequencing

Two pools of RNA samples were equimolarly mixed to build each of the four libraries, two for each species. One library with seven samples and another with six for *R*. *montenegrensis* were built, as well as two libraries with six samples each for *R*. *robustus*, making these libraries independent biological replicates of each species. These libraries were constructed using the TruSeq^®^ RNA Sample Prep kit v2 (Illumina) following the protocol suggested by the manufacturer. Runs were performed in the Illumina HiSeq2500 platform with 2 x 100 bp paired-end reads in the Laboratory for Functional Genomics Applied to Agriculture and Bioenergy of the Luiz de Queiroz College of Agriculture (ESALQ) at the University of São Paulo (USP) in Brazil.

### *De novo* transcriptome assembly

All reads were trimmed for quality using Phred quality scores and filtered for length using the SeqyClean program, which is available at https://bitbucket.org/izhbannikov/seqyclean. The quality filters were set to “qual 20 20” and “minimum_read_length 50”. SeqyClean was also used to remove any remaining adapter sequences in the reads.

Independent *de novo* assemblies were performed for each species with reads from their two replicas. To optimize contig assembly, the filtered reads were normalized using the *insilico_read_normalization*.*pl* tool, which is included in the Trinity package [27]. In order to avoid redundancy, the maximum read coverage was set to 60. The filtered and normalized reads were assembled using the Trinity software [27]. The quality and completeness of the assemblies were evaluated using BUSCO (Benchmarking Universal Single-Copy Orthologs) [28]. For this analysis, all transcripts of each assembly were compared with a set of single copy orthologs of Arthropoda.

### Coding region (CDS) prediction and functional annotation

The TransDecoder tool included in the Trinity package was used to predict the coding regions based on open reading frames (ORFs) for each species’ assembly, retaining coding regions that were over 100 amino acid residues. We compared the predicted coding regions against the non-redundant (NR) protein GenBank database and the *R*. *prolixus* genome [29], available at VectorBase (https://www.vectorbase.org/) based on similarity using Blast program with an E-value threshold of 10^−6^. The functional annotation was carried out using Blast2GO program [30]. This analysis allows us to associate the CDS with the Gene Ontology (GO) terms that describe gene product attributes considering three different ontologies, Biological Process, Molecular Function and Cellular Component. The annotations were analyzed in the WEGO software (http://wego.genomics.org.cn/cgi-bin/wego/index.pl) to produce the distribution of GO terms at level 2 for each species’ transcriptome. An enrichment analysis of the highly differentiated contigs on the GO terms was performed using the TopGO program [31], retaining the significantly enriched contrasts at the 0.05 significance level after FDR correction using the method of Benjamini and Hochberg [32].

### Sequence alignment and Single Nucleotide Polymorphisms (SNP) calling

To avoid redundancy in the mapping of the *R*. *robustus* assembly, only the largest isoforms from each unigene detected during the assembly process were kept. This way, a single transcript was selected for each set of isoforms (unigene). The filtered reads from each species were mapped against the *R*. *robustus* assembly, which was taken as a reference transcriptome using the default settings of the Bowtie2 program [33]. The files containing the mapped reads were converted into the pileup format using the mpileup tool of the Samtools program, version 0.18 (http://samtools.sourceforge.net). The software VarScan 2 was then used to call the intra and interspecific SNPs [34] by means of the mpileup2snp command. The analyses relied on a minimum coverage of 42, a minimum mapping quality score of 30, and a Phred quality score greater than or equal to 30. (commands used in each step are available in the S1 Table).

In order to select a group of candidate transcripts that showed the greatest level of divergence between the two species, the index of interspecific differentiation (D) [35] was estimated based on the allele frequencies estimated by VarScan 2. The D variable, which is defined as the absolute value of the difference between the allele frequencies of an SNP from *R*. *montenegrensis* and *R*. *robustus* (D = │FRm—FRr│), was calculated. Furthermore, the average D value was estimated including the SNPs from a particular transcript ($\bar{\text{D}}$) [35]. In the search for possible candidate genes involved in the process of speciation, transcripts including only fixed variants ($\bar{\text{D}}$ = 1) were used.

### Test of selection

The ratio of non-synonymous and synonymous rates (Ka/Ks) was used to identify potential transcripts evolving under positive selection. Ka/Ks > 1 indicates signal of natural selection, Ka/Ks < 1 purifying selection and Ka/Ks = 0 neutrality [36]. This analysis was carried out through a search for ortholog CDSs using best hit reciprocal Blast strategy and an e-value threshold of 10^−12^. The pairs of ortholog CDSs were translated into protein, and aligned using muscle algorithm [37] and then back translated to DNA using software TranslatorX [38]. The Ka/Ks values were estimated using the model selection criterion implemented in the software Kaks_Calculator [39]. Pair of CDSs with Ks ≥ 1 may be paralogs, so they were removed from the analysis.

## Results and discussion

### *De novo* transcriptome assembly

The four RNA libraries (two replicas per species) generated 131,250,862 paired-end 100 base pairs (bp) reads, an average of 32.8 million reads per library. The produced reads were filtered by quality, resulting in 124,601,286 paired-end reads and an average of more than 31 million reads per replica in the two species studied (Table 1). RNA-Seq data were *de novo* assembled per species using Trinity leading to the production of 53,042 and 56,756 contigs for *R*. *montenegrensis* and *R*. *robustus* assemblies, respectively, with similar N50 of about 2,100. However, N50 is not an appropriate measure of quality for transcriptome assemblies because it is dependent on the transcript lengths that vary between species and even between tissues. We therefore used the BUSCO program to evaluate the completeness of the assemblies from its gene content [28]. BUSCO showed that these assemblies had more than 80% (1,320 complete single-copy orthologs of 2,675 total BUSCO groups searched in *R*. *montenegrensis* and 1,281 of 2,675 in *R*. *robustus)* of complete Arthropods single copy orthologs, which is consistent with what was expected for transcriptomes (S1 Fig).

**Table 1:** Summary of sequencing and assembly statistics based on cephalic tissue from males of *R*. *montenegrensis* (Rm) and *R*. *robustus* (Rr).

	Rm	Rr
Total reads	32,502,355	33,123,076
Reads filtered by quality	30,772,029	31,528,614
Reads after normalization x 2 reads	6,543,879	7,296,681
Total trinity unigenes	53,042	56,756
Total trinity transcripts	64,952	70,894
Percent GC	34.66	34.68
N50	2,083	2,134
N50 unigenes	1,572	1,585
Contigs larger than 1000 bp	19,838	21,652
Contigs larger than 2000 bp	9,903	10,918
Mean (bp)	477	480
Median (bp)	1,046	1,057
Total assembled bases	67,920,294	74,955,989
Largest contig (bp)	32,076	30,264

### Coding Sequence (CDS) prediction and functional annotation

TransDecoder identified 27,150 CDSs in the *R*. *montenegrensis* assembly and 28,965 in the *R*. *robustus* assembly. Out of those, 17,207 and 18,806 complete coding regions of *R*. *montenegrensis* and *R*. *robustus* were predicted, respectively.

A similarity search with BLASTx, identified 16,057 coding regions in *R*. *montenegrensis* and 16,781 coding regions in *R*. *robustus* with a significant hit against the NR database. Out of these hits, 12% had the best hit associated to *Tribolium castaneum* (Coleoptera) sequences for both species (S2 Fig). Of the total sequences with significant hits against the NR, 10,442 *R*. *montenegrensis* sequences and 10,828 *R*. *robustus* sequences had functional annotation associated with Gene Ontology terms. In addition, 2,412 coding regions of *R*. *montenegrensis* and 2,447 coding regions of *R*. *robustus* were found to be related to enzyme codes in the KEGG database. The annotation against the genome of *R*. *prolixus* revealed that 22,871 CDSs of head transcriptomes of *R*. *montenegrensis* and 24,246 of *R*. *robustus* had significant hits with CDSs predicted from this genome.

**Fig 1:**
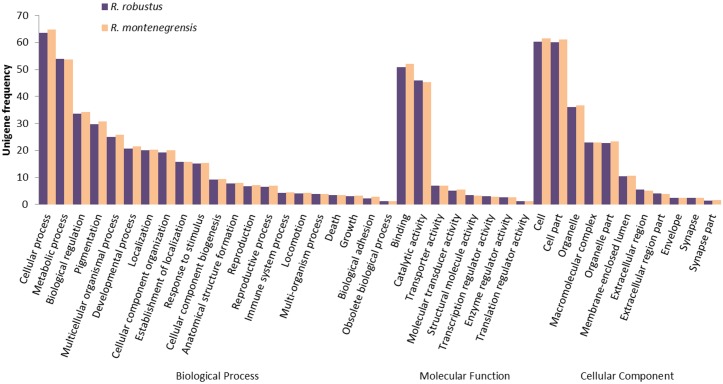
Gene Ontology level 2 terms with frequencies greater than 1% found for coding regions of unigenes derived from the head transcriptomes of males of *R*. *montenegrensis* and *R*. *robustus*.

Using the WEGO software, the annotated coding regions were distributed into 55 different level-2 GO categories, related to three main classes: Biological Processes (18), Molecular Function (24), and Cellular Component (13). Cellular Process was the most represented category in Biological Process, being associated with 64.9% of the coding regions of *R*. *montenegrensis* and 63.5% of the coding regions of *R*. *robustus*. Most of the annotated transcripts were found to be associated with the Binding category in Molecular Function, with 52% of the transcripts in *R*. *montenegrensis* and 50.9% in *R*. *robustus*, whereas the Cell Part category was associated with 61.5% of the transcripts in *R*. *montenegrensis* and 61.2% in *R*. *robustus*, being the most represented in the Cellular Component class (Fig 1).

An enrichment analysis in GO terms revealed eight terms that were significantly enriched in the transcriptome which were found to be associated with the clathrin-coated endocytic vesicle membrane (Table 2). Proteins associated with this GO term have also been found in *Drosophila* [40] and in the transcriptome of the digestive tract of *R*. *prolixus* [41]. It is a multimeric coat protein with intracellular sorting roles and influences on mitosis, which is linked to a variety of functions in human health such as infection disease and metabolism [42].

**Table 2:** Enrichment analysis of the GO terms of the most divergent unigenes ($\bar{\text{D}}$ = 1). P-values were corrected using the method proposed by Benjamini and Hochberg [32].

GO Term	Category Name	Main Category	p-value
GO:0030128	clathrin coat of endocytic vesicle	Cellular Component	0.01278887
GO:0030122	AP2 adaptor complex	Cellular Component	0.01278887
GO:0045334	clathrin-coated endocytic vesicle	Cellular Component	0.02489061
GO:0030669	clathrin-coated endocytic vesicle membrane	Cellular Component	0.02489061
GO:0030132	clathrin coat of coated pit	Cellular Component	0.02489061
GO:0030666	endocytic vesicle membrane	Cellular Component	0.0423901
GO:0030125	clathrin vesicle coat	Cellular Component	0.0423901
GO:0005905	coated pit	Cellular Component	0.0423901

### Test of selection

**Fig 2:**
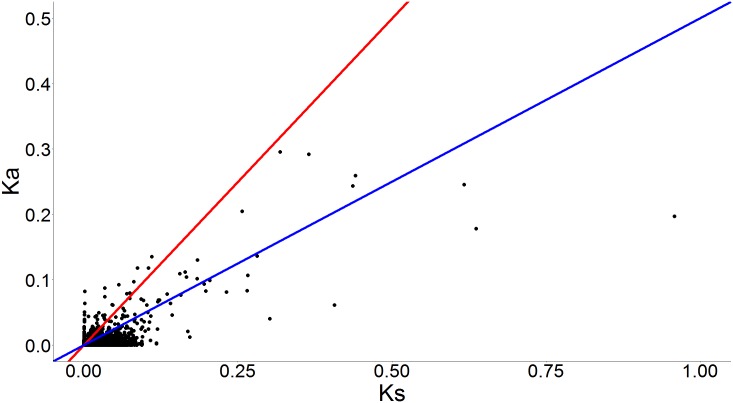
Scatter plot of non-synonymous (Ka) and synonymous (Ks) rates among ortholog CDSs expressed in head transcriptomes of males of *R*. *montenegrensis* and *R*. *robustus*. Ka/Ks = 1 and and Ka/Ks = 0.5 are shown in red and blue lines, respectively.

Out of the 6,951 pairs of ortholog CDSs, we identified 549 pairs with a Ka/Ks > 1 (Fig 2), which suggests that they have potentially evolved under positive selection. Several of these genes with signs of positive selection are related to proteins involved in host haemostatic defences, which comprise antihaemostatic, haemolytic, vasodilator and anticoagulant properties [43–45] (Table 3). Thus, they are most likely to be involved in rapid adaptation to host responses and may be potentially relevant in the search for genes that separate different *Rhodnius* species. We chose to highlight some relevant protein classes, even though they were not significantly enriched in the tests performed, because the majority of genes potentially under positive selection lack association with Gene Ontology terms, limiting any enrichment tests.

**Table 3:** Summary of functional annotation and inferred evolutionary rates (Ka, Ks and Ka/Ks) of unigenes in head transcriptomes of *R*. *montenegrensis* and *R*. *robustus*.

Annotation	Function	Ka	Ks	Ka/Ks
Salivary kazal-type proteinase inhibitor	Anticoagulant, vasodilator and anti-microbial activities [60].	0.00539	1.08 x 10^−4^	50
Salivary lipocalin	Transportation of hydrophobic compounds in aqueous biological fluids, and as a binding protein sex pheromone [57].	0.07865	6.9827 x 10^−2^	1.12635
Odorant-binding protein rproobp4 precursor	Precursor odor binding protein and / or pheromones [59].	0.002812	5.62 x 10^−5^	50
Hemolysin-like secreted salivary protein 2	Poration toxin and is often described in the microorganism [53].	0.011252	2.25 x 10^−4^	50
Heme-binding protein	Prevents heme-induced oxidative damage to lipophorin [56].	0.028048	5.61 x 10^−4^	50
Diptericin	Antibacterial peptide [61].	0.046749	9.35 x 10^−4^	50
Nitrophorin 3b	Antihaemostatic [58].	0.117836	1.12417	0.719394

Ka: non-synonymous; Ks: synonymous; Ka/Ks. ratio of non-synonymous to synonymous rates.

A class of proteins well represented in the list of rapidly evolving genes are: proteinases and Kazal-type inhibitor of proteinases which have been found to evolve rapidly in some species [46] and are common in salivary transcriptomes of various insects, including *Aedes aegypti*, *R*. *prolixus*, *T*. *infestans*, *T*. *matogrosensis e P*. *megistus* [41, 47–50]. Proteinase inhibitors have a role in controlling host proteinases in plant feeding insects [51], as well as anticoagulant, vasodilator and anti-microbial activities in salivary glands of bloodsucking insects [48, 49]. Proteinases in general, on the other hand, had an important role into the transformation of Triatominae from sap eating bugs to blood sucking insects, which is rich in proteins [52].

There are other proteins with high evolutionary rates in the head transcriptome of *Rhodnius* which have been associated with processing and digesting of blood proteins, such as heme oxigenase, heme binding proteins and Hemolysin-like secreted salivary protein, which is described in microorganisms as a pore-forming toxin [53]. Though the function of this latter protein in triatomine is not well known, some authors believe it may be involved in the lysis of red blood cells [54], having been also found in *P*. *megistus*, *T*. *matogrosensis* e *R*. *prolixus* [49, 50]. Heme-binding proteins on the other hand can bind to nitric oxide to prevent platelet aggregation and vasodilation promoting vertebrate host [55]. These proteins inhibit the peroxidation of heme-dependent primary lipoprotein hemolymph in *R*. *prolixus* and that prevents oxidative damage induced by heme throughout the blood suction period [56].

We also identified salivary lipocalins which belong to a family of prokaryote and eukaryote proteins characterized by having a highly conserved structure that allows their proteins to carry hydrophobic compounds, such as lipids, in aqueous biological fluids. Several antihemostatic proteins common to triatomine were found to belong to this family, such as nitric oxide lipocalins found In *R*. *prolixus*, which has been connected with the vasodilator and platelet aggregation inhibitory function in vertebrate hosts [49]. Because of their hydrophobic affinity, some lipocalins were also described as binding protein sex pheromone [57]. Other lipocalins, such as *Nitrophorin 3b*, has also commonly been found in the hematophagous arthropods and their transcriptomes, such as in *T*. rubida, *P*. *megistus*, and *R*. *prolixus* [41, 49, 50, 58]. This protein is a type of lipocalin that binds nitric oxide and has antihemostatic action.

Another set of relevant proteins we identified are related to chemosensory perception. We have identified several Odorant Binding Proteins, as well as Chemosensory Binding Proteins, which have been associated to odor identification such as host or pheromone sensing, such as the precursor of Odorant-binding protein (OBP) RproOBP4 [59]. We should point out that the transcriptomes here produced contain not only antennal tissues, but also salivary tissues, so it is possible that these OBPs may have a different role as well, since in the gut of *R*. *prolixus* some OBPs are related to the transport of nutrients or other molecules involved in coordinating the intestinal physiological function [41].

### SNP calling

**Fig 3:**
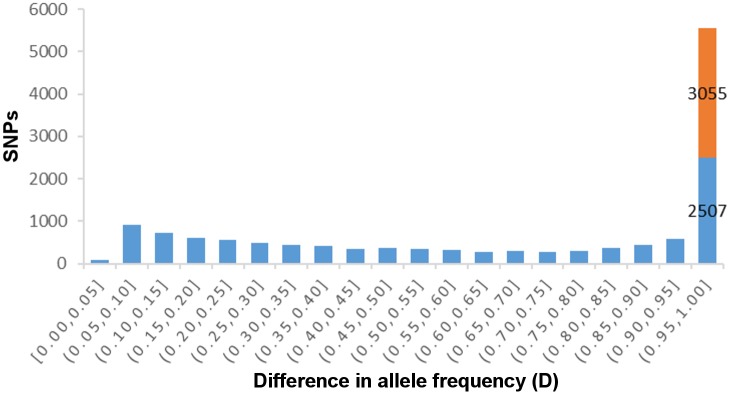
Distribution of absolute allele frequency differences (D) of the 13,696 shared SNPs between *R*. *montenegrensis* and *R*. *robustus*. We highlight in red the class of SNPs with D = 1.

**Fig 4:**
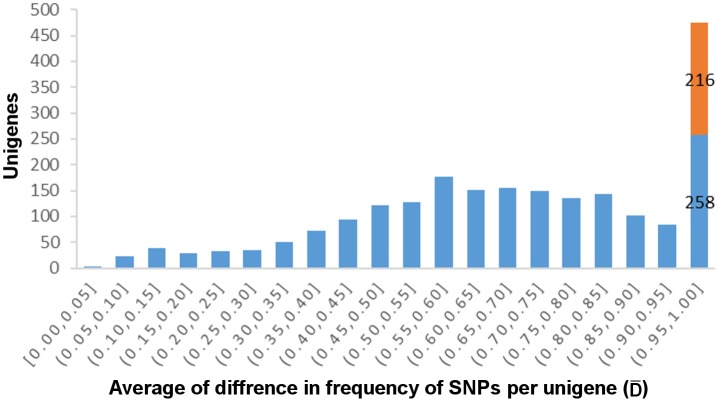
Absolute allele frequency difference averages of shared SNPs of *R*. *montenegrensis* and *R*. *robustus* per transcript ($\bar{\text{D}}$). We highlight in red the class of SNPs with $\bar{\text{D}}$ = 1.

**Fig 5:**
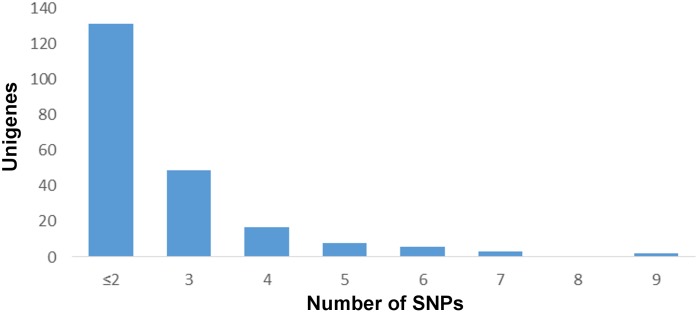
Distribution of number of SNPs per contig that are 100% fixed for different alleles between *R*.*montenegrensis* and *R*. *robustus*.

A comparison of the assemblies of both species identified 13,695 SNPs in 2,196 different contigs. Out of these SNPs, 8,350 are fixed in *R*. *montenegrensis* and 7,888 in *R*. *robustus*. Using the frequency of each SNP, a distribution of the differentiation index (D) reveals that the SNPs are in general homogeneously distributed across almost all D classes, with the exception of the most differentiated class. This class alone harbors 5,552 SNPs, i.e, over 40% of the total number of SNPs found in the analysis of *R*. *montenegrensis* and *R*. *robustus*. That indicates a great number of SNPs that were fixed, or nearly fixed, for different nucleotides between the species. Furthermore, 3,055 of these had a D of 100%; in other words, the different species were fixed for different nucleotides (Fig 3). Considering the average index ($\bar{\text{D}}$) by contig, which averages the D values of all of the SNPs in each contig, 558 contigs, out of a total of 2,196, had a $\bar{\text{D}}$ value above 90%, and 216 showed a $\bar{\text{D}}$ of 100% (Fig 4), 85 of them with three or more SNPs (Fig 5), again indicating that all SNPs in these contigs are fixed for different nucleotides between the species studied (Figs 4 and 5).

### Speciation and fixed SNPs

There are morphological and genetic differences among the two species here studied which led to their recognition as separate species [62]; nevertheless, these differences were previously considered not to warrant them separation at the species level by Abad-Fanch [18]. The few morphological differences identified may be a consequence of recent divergence, however, the current SNP analysis shows that 5,562 (40.6%) of the SNPs identified were fixed between the species (Fig 2). It has been suggested that only a few genes in general are involved in the beginning of the speciation process. As time goes by, drift and linkage disequilibrium increase the number of genes with fixed differences, which may extend to whole chromosomes and even the whole genome [63, 64]. Thus, recently diverged species would show only a few fixed SNPs and, with new barriers to reproduction, more differences would be fixed in these genomes. We would expect that if *R*. *montenegrensis* and *R*. *robustus* belonged to the same species, we would see a distribution of D close to normal, but with values closer to zero, since they would share many polymorphisms. However, the results show that more than 30% of the SNPs were fixed for different nucleotides in these two species, a finding which reflects more than 7,000 SNPs with an average of D > 95% (Fig 3). The fact that there are multiple SNPs fixed in the same contig is particularly interesting, since it might reflect the occurrence of strong selection favoring the fixation of variants in different species, or simply a consequence of the long time that has passed since these populations diverge. This substantial number of fixed polymorphisms suggests a clear separation of lineages and reinforces the validity of *R*. *montenegrensis*, contrary to what was suggested by Abad- Franch *et al*. [4, 18] that the latter species would be a variety of *R*. *robustus*. Furthermore, these data provide a long list of potential markers that may be used to differentiate *R*. *montenegrensis* from *R*. *robustus* that need to be corroborated in more populations across the species’ distribution.

### Candidate markers for identifying species of *Rhodnius*

Some species in the genus *Rhodnius* are difficult to identify using morphological characters [65, 66], especially when the samples are not analyzed by expert taxonomists [66, 67]. To solve this problem, some authors have suggested the use of molecular markers, such as sequences from the mitochondrial gene cytochrome b, which was claimed to be effective at separating several species in the genus [16, 68, 69]. However, even though this marker differentiated *R*. *montenegrensis* and *R*.*robustus* [17], the genetic distance between these two species was considered to be too low to warrant them separate species status [18]. Several authors have already questioned the efficacy of a “one gene solves all taxonomic problems” [70, 71], so, even though mitochondrial genes have had tremendous success to help with taxonomic hurdles in some taxa, even Hymenoptera [72, 73], its efficacy is not universal [71, 74, 75], particularly when you have recent divergence of large populations sizes with continuous gene flow. In that case, we may need to retort to several nuclear genes, particularly those with faster evolutionary rate that may have a better chance of tracking the species differences. Considering the method used to produce these assemblies, we found a number of contigs with high level of differentiation between *R*. *montenegrensis* and *R*. *robustus*, among which, 11 that were fixed between these species and potentially evolved under positive selection (Ka/Ks > 1). Interestingly, in these contigs we found genes coding for proteins involved in cell metabolism as well as other related to host interaction response (Table 4), which makes them potentially useful for identifying *R*. *montenegrensis* from *R*. *robustus* and perhaps might prove to be useful for speciation studies [76] of other species in the genus, as well.

**Table 4:** Unigenes highly differentiated and potentially evolved under positive selection (Ka/Ks > 1) in head transcriptomes of *R*. *montenegrensis* and *R*. *robustus*.

Contig RR	Annotation	Function	SNP	$\bar{\text{D}}$	Ka	Ks	Ka/Ks
c10228_g1_i1	PREDICTED: zinc finger CCHC-type and RNA-binding motif-containing protein 1-like	Binds to RNA, being involved in many stages of its metabolism [77]	4	1	3.94 x 10^−3^	7.90 x 10^−5^	50
c10808_g1_i1			6	1	1.86 x 10^−2^	3.7 x 10^−4^	50
c11678_g1_fi1	phosphopentothenoylcysteine decarboxylase	Acts in biosynthesis of coenzyme A [78, 79].	4	1	6.33 x 10^−3^	1.3 x 10^−4^	50
c13526_g1_i2			4	1	8.62 x 10^−3^	5.28 x 10^−3^	1.63269
c13603_g1_i1	nucleolar phosphoprotein-like	Ribosomal protein encoding and transport, control of centrosome duplication [80].	6	0.9993	1.59 x 10^−2^	1.09 x 10^−2^	1.45733
c41453_g1_i1	PREDICTED: uncharacterized protein LOC101741012 isoform X1		2	0.9969	8.44 x 10^−3^	1.7 x 10^−4^	50
c13063_g1_i1	hypothetical protein TcasGA2_TC012103		7	0.9959	3.63 x 10^−3^	7.30 x 10^−5^	50
c10204_g1_i1	PREDICTED: heme oxygenase 1-like	Catalyzes the degradation of heme. Important for digestion of the blood meal [49, 50].	10	0.9949	6.67 x 10^−3^	1.3 x 10^−4^	50

RR: *Rhodnius robustus*; SNP: Single Nucleotide Polymorphisms;

$\bar{\text{D}}$: Average of SNP allele frequency differences between species per contig; Ka: rate of non-synonymous substitutions; Ks: rate of synonymous substitutions; Ka/Ks. ratio of non-synonymous and synonymous rates.

## Conclusion

The study on head tissues transcriptomes from *R*. *montenegrensis* and *R*. *robustus* generated thousands of interesting genes that shed some light in the evolution and divergence of these species, as well as helped establish potential targets for Triatominae in general, as these data can be combined with previous transcriptomes available for species in this subfamily. The contig analysis generated thousands of polymorphic SNPs, a great number of them fixed for different nucleotides in *R*. *robustus* and *R*. *montenegrensis*, which suggests that these two species have a greater level of divergence than previously considered and might corroborate morphological and morphometric studies that differentiate them. Moreover, these results provide a plethora of genes potentially evolving under positive selection that might be relevant in the quest of finding markers that could not only distinguish *R*. *montenegrensis* and *R robustus* but perhaps be very useful for studies among other species in the genus and even across genera in Triatominae.

## Supporting information

S1 Fig BUSCO results. 80% (1,320 complete single-copy orthologs of 2,675 total BUSCO groups searched in R. montenegrensis and 1,281 of 2,675 in R. robustus) of complete arthropods single copy orthologues, 8% fragmented and 12% missing of complete arthropods single copy orthologues in both species.
(TIF)

S2 Fig *R*. *montenegrensis* and *R*. *robustus* with a significant hit (%) against species of NR database. BLASTx, identified 16,057 coding regions in *R*. *montenegrensis* and 16,781 coding regions in *R*. *robustus* with a significant hit against the NR database.
(TIF)

S1 Table Commands used in each softwares. (PDF)
