# Supplementary figures and images for: Differential transcriptome analysis supports Rhodnius montenegrensis and Rhodnius robustus (Hemiptera, Reduviidae, Triatominae) as distinct species
Source: PLoS One. 2017 Apr 13;12(4):e0174997. doi: 10.1371/journal.pone.0174997 (PMC5390988; doi:10.1371/journal.pone.0174997)

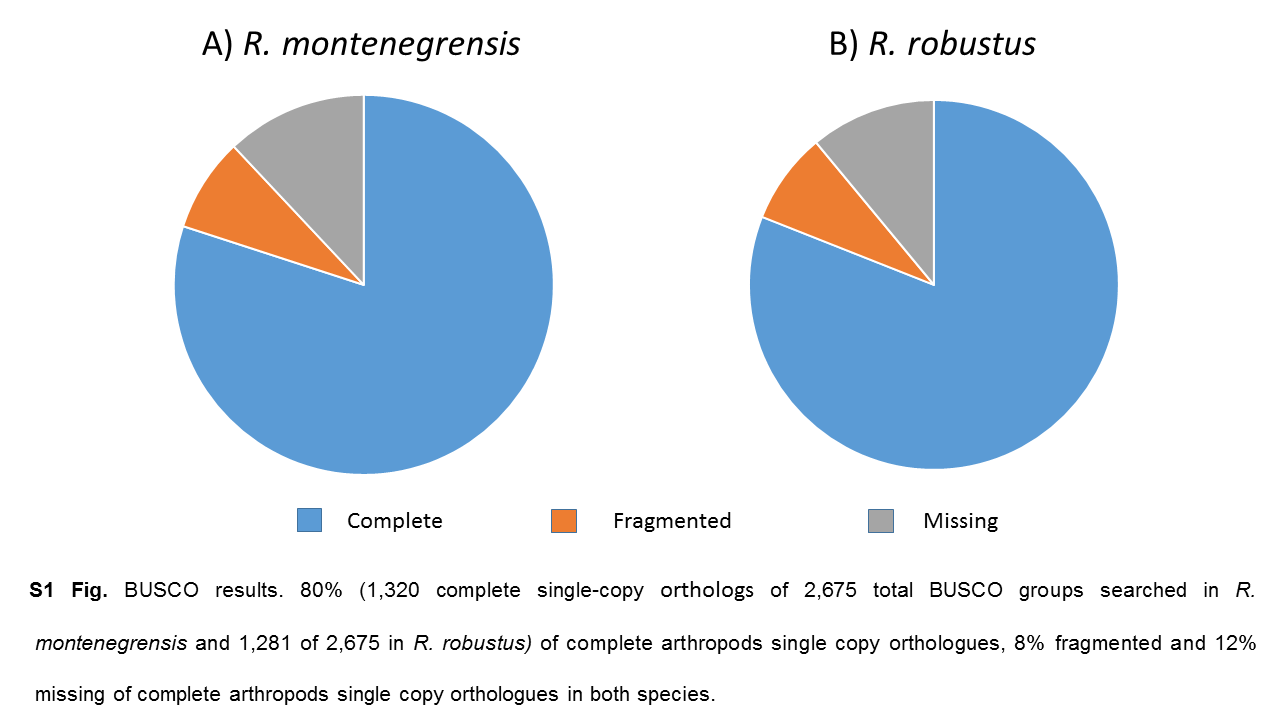

Supplement: S1 Fig — 80% (1,320 complete single-copy orthologs of 2,675 total BUSCO groups searched in R. montenegrensis and 1,281 of 2,675 in R. robustus) of complete arthropods single copy orthologues, 8% fragmented and 12% missing of complete arthropods single copy orthologues in both species. (TIF) [file pone.0174997.s001.TIF]

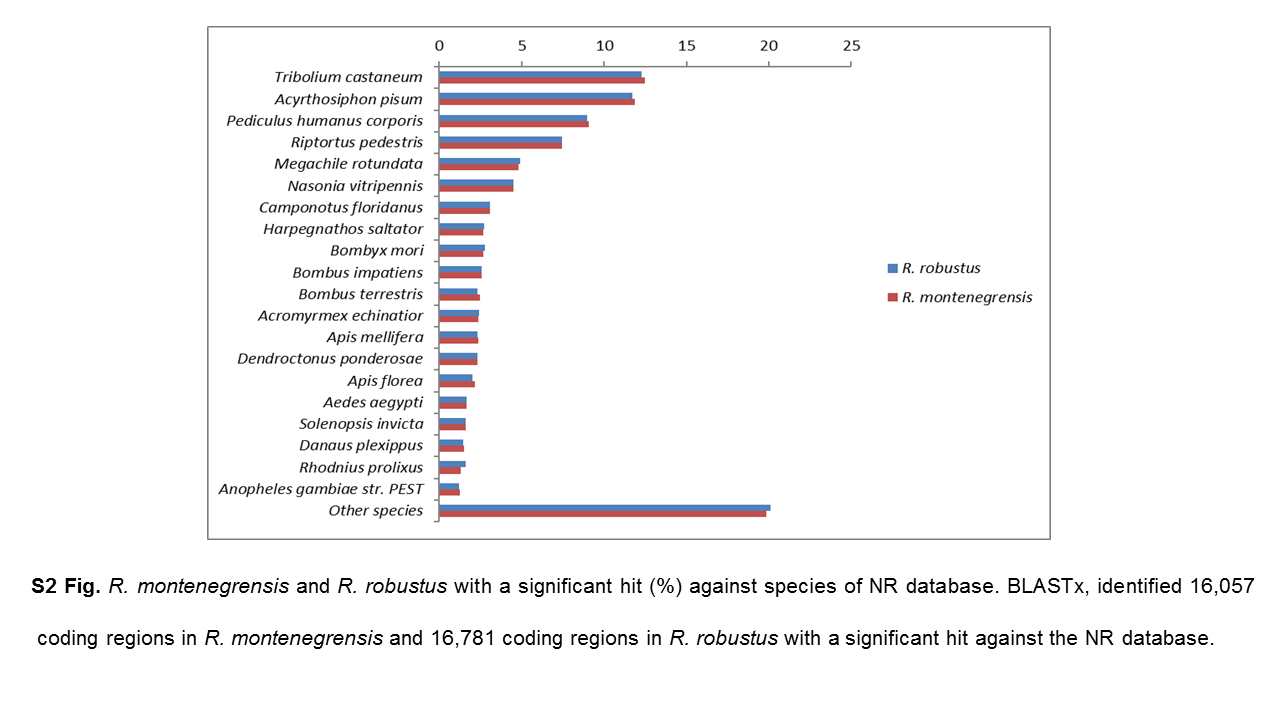

Supplement: S2 Fig — BLASTx, identified 16,057 coding regions in R. montenegrensis and 16,781 coding regions in R. robustus with a significant hit against the NR database. (TIF) [file pone.0174997.s002.TIF]
