# Supplementary material for: Differential transcriptome analysis supports Rhodnius montenegrensis and Rhodnius robustus (Hemiptera, Reduviidae, Triatominae) as distinct species
Source: PLoS One. 2017 Apr 13;12(4):e0174997. doi: 10.1371/journal.pone.0174997 (PMC5390988; doi:10.1371/journal.pone.0174997)
Supplement: S1 Table — (PDF) [file pone.0174997.s003.pdf]

S1 Table Commands used in each Softwares

Softwares	Commands
**Quality filter**	./seqyclean -qual 20 20 -minimum_read_length 50 --fastq -1 [input_file_PE_1] -2 [input_file_PE_2] -o [output_prefix]
**Normalization**	./insilico_read_normalization.pl --seqType fq --JM 100G --left [input_filtered_file_PE_1] --right [input_filtered_file_PE_2] --pairs_together --KMER_SIZE 25 --max_cov 60 --output [output_dir]
**Assembly**	./Trinity --seqType fq --left [input_filtered_normalized_file_PE_1] --right [input_filtered_normalized_file_PE_2] --min_contig_length 200 --output [output_dir]
**Quality assessment**	python3 ./BUSCO_v1.1b1.py -o [dir_output] -in [input_trinity_assembly] -l [single_copy_orthologs_arthropoda] -m trans --ev 0.000001 -c 20
**Mapping**	./bowtie2-build [Rr_trinity_assembly] [bt2_index_prefix]<br>./bowtie2 -p 10 -x [Rr_bt2_index_prefix] -1 [input_filtered_file_PE_1] -2 [input_filtered_file_PE_2] -S [output_sam]<br>./samtools view -bS [input_sam] > [output_bam]<br>./samtools sort 2 [input_bam] [output_sorted_prefix]
**SNP calling**	samtools faidx [trinity_assembly]<br>samtools mpileup -d 80000000 -q 30 -f [Rr_trinity_assembly_faidx] [output_sorted_Rm_sample1_bam] [output_sorted_Rm_sample2_prefix] [output_sorted_Rr_sample2_prefix] [output_sorted_Rr_sample2_prefix] | java -jar ./VarScan_v2.3.6.jar mpileup2snp --min-coverage 42 --min-reads2 3 --min-avg-qual 30 --min-var-freq 0.01 --min-freq-for-hom 0.99 --p-value 0.01 --strand-filter 1 --output-vcf 1 > [output_file]
